# Life history and demographic determinants of effective/census size ratios as exemplified by brown trout (*Salmo trutta*)

**DOI:** 10.1111/j.1752-4571.2012.00239.x

**Published:** 2012-01-23

**Authors:** Dimitar Serbezov, Per Erik Jorde, Louis Bernatchez, Esben Moland Olsen, Leif Asbjørn Vøllestad

**Affiliations:** 1 Centre for Ecological and Evolutionary Synthesis (CEES), Department of Biology, University of OsloBlindern, Oslo, Norway; 2 Institute of Marine ResearchFlødevigen, Norway; 3 Institut de Biologie Intégrative et des Systèmes (IBIS), Pavillon Charles-Eugène Marchand, Université LavalQuébec, QC, Canada

**Keywords:** animal mating/breeding systems, conservation genetics, fisheries management, life history evolution

## Abstract

A number of demographic factors, many of which related to human-driven encroachments, are predicted to decrease the effective population size (*N*_e_) relative to the census population size (*N*), but these have been little investigated. Yet, it is necessary to know which factors most strongly impact *N*_e_, and how to mitigate these effects through sound management actions. In this study, we use parentage analysis of a stream-living brown trout (*Salmo trutta*) population to quantify the effect of between-individual variance in reproductive success on the effective number of breeders (*N*_b_) relative to the census number of breeders (*N*_i_). Comprehensive estimates of the *N*_b_*/N* ratio were reduced to 0.16–0.28, almost entirely due to larger than binomial variance in family size. We used computer simulations, based on empirical estimates of age-specific survival and fecundity rates, to assess the effect of repeat spawning (iteroparity) on *N*_e_ and found that the variance in lifetime reproductive success was substantially higher for repeat spawners. Random family-specific survival, on the other hand, acts to buffer these effects. We discuss the implications of these findings for the management of small populations, where maintaining high and stable levels of *N*_e_ is crucial to extenuate inbreeding and protect genetic variability.

## Introduction

The effective population size (*N*_e_) is arguably one of the most important parameters in evolutionary and conservation biology as it determines the rate of genetic drift, the rate of loss of diversity of neutral alleles and the rate of increase of inbreeding experienced by an isolated population (Charlesworth and Willis 2009). This is because it establishes the relative evolutionary importance of stochastic (random genetic drift) and directional (migration and selection) factors and is thus closely being linked to genetic diversity (Crow and Kimura 1970). Genetic variation is the raw material for evolutionary change, allowing populations to evolve as a response to various environmental challenges (Frankham 1996), be they natural or anthropogenic, and its maintenance is therefore a fundamental concern in conservation biology. To be able to properly manage wild resources, genetic and evolutionary considerations have to be integrated into management practices (Young 2004; Waples and Naish 2009).

Because the magnitude of drift is inversely proportional to the effective size (e.g., Crow and Kimura 1970), small natural populations or populations experiencing fragmentation and loss of connectivity, often the focus of conservation programmes, are vulnerable to the random loss of genetic variation. Knowledge of the effective size is therefore particularly important in such cases (Lande and Barrowclough 1987; Nunney and Elam 1994). Anthropogenic activities have recently resulted in many wild populations being seriously reduced in size, leading to significant reductions in *N*_e_ (Frankham et al. 2010). Supportive breeding programmes may also be disadvantageous from a conservation genetics perspective if the individuals released into the wild originate from captive populations with small *N*_e_ values (Ryman and Laikre 1991). Also, the efficiency of natural selection is directly proportional to *N*_e_ because genetic drift is weaker when *N*_e_ is large, and this will determine the fate of natural populations (Charlesworth 2009).

Despite its fundamental importance in conservation genetics, it has been notoriously difficult to obtain reliable *N*_e_ estimates in the wild. Accurate ecological *N*_e_ estimation requires quantification of a number of demographic parameters that are difficult to obtain from natural populations in most species. Examples of these include accounting for spatial substructuring and overlapping generations. In addition, a variety of factors related to its interpretation complicate its application in wild populations. The ratio between the effective population size to the number of sexually mature adults in a population (census size), the *N*_e_*/N* ratio, is of great potential interest. Different groups of organisms, however, seem to be characterized by different *N*_e_*/N* ratios, leading to confusion regarding the magnitude and the meaning of this parameter (Nunney 1993; Frankham 1995). Also, if there is a relationship between *N* and *N*_e_, variable population size can have a profound influence on long-term *N*_e_ because effective size for a series of generations is approximately the harmonic mean of the single generation *N*_e_ (Wright 1938). This effect would be particularly relevant in populations of conservational concern, where an apparently healthy census population may mask small *N*_e_ (Nunney and Elam 1994; Frankham 1995).

The most important factors responsible for reducing *N*_e_ below the number of sexually mature adults in the population is a larger than binomial variance in individual reproductive success, unequal sex ratio and temporal fluctuations in population size (Wright 1938). Polygamous mating systems would result in high variance in reproductive success that in turn leads to low effective population size (Nunney 1993; Ardren and Kapuscinski 2003). In real populations, the variance of progeny number per family generally exceeds the mean progeny number in both sexes (Crow and Kimura 1970), so that the effective number of individuals of either sex is less than the actual number present in any generation. However, as generations overlap, there is no easy way of quantifying this effect on the per-generation *N*_e_ (Waples 2010). Estimates of reproductive success are commonly obtained using seasonal measures, but these primarily estimate the effective number of breeders per year (*N*_b_) rather than the effective population size per generation (*N*_e_). The *N*_b_ values are however only useful in estimating *N*_e_ if it can be assumed that female and male fecundity is age-independent (Nunney 1993). Because *N*_e_ is based on lifetime reproductive success, such estimates are much more difficult to obtain for wild populations of iteroparous organisms. Moreover, to estimate generational *N*_e_ from demographic data, parents and their offspring should be counted at the same life stage. Family-correlated survival is expected to reduce *N*_e_ further compared to random survival (Crow and Morton 1955; Hedgecock 1994). Little is currently known about the relationships between life history plasticity, demographic change and *N*_e_ (Lande and Barrowclough 1987; Nunney 1993), partly due to scarcity of the ecological data required for these analyses.

Salmonid fishes exhibit a wide diversity of breeding systems (Fleming 1998; Fleming and Reynolds 2004), and even different populations of the same species living under different environmental conditions might have evolved different life history tactics and can thereby exhibit different breeding behaviours (Gross 1991). Both sexes in salmonids show high variance in reproductive success (Fleming 1998; Garant et al. 2001; Serbezov et al. 2010). However, reduced variance in individual reproductive success at low breeder abundance (genetic compensation) can lead to an increase in *N*_e_ and might be a realistic aspect of salmonid breeding systems (Ardren and Kapuscinski 2003; Frasier et al. 2007). Understanding the relationship between *N*_e_ and the mating system is therefore important in allowing comparisons between populations in conservation contexts (Nunney 1993). Proper management of such populations should account for those factors that may potentially strongly impact (i.e., reduce) the *N*_e_ and thus genetic variance. Indeed, in addition to climate change, salmonid fishes are exposed to a large number of human encroachments such as harvesting and fragmentation leading to reduced connectivity. Many of these encroachments influence the breeding system, levels of gene flow, population structure and overall fitness, thus impacting *N*_e_ and the overall level of genetic variation. To manage such populations, it is necessary to know which factors most strongly impact *N*_e_ and how to mitigate these impacts through management actions. In this study, we use extensive demographic and genetic data on a brown trout (*Salmo trutta*) population to quantify the effects of variation in reproductive biology on *N*_b_ and *N*_e_. We use empirical estimates of age-specific survival (Carlson et al. 2008) and age- and sex-specific fertility (Serbezov et al. 2010) in an individual-based simulation model to estimate the variance in lifetime reproductive success and obtain a generational *N*_e_ estimate. Having this information, it is possible to evaluate what kind of population structure best maintains high and stable levels of *N*_e_ and thus help make informed management decisions.

## Materials and methods

### Background

The study site is a small forest stream, Bellbekken, in South-East Norway (N: 61°15′, E: 11°51′) that was sampled extensively during the period 2002–2007 (see Olsen and Vøllestad 2003; Carlson et al. 2008; Serbezov et al. 2010 for detailed information). Compared to nearby rivers, Bellbekken has limiting growth conditions for fish and is seldom, if ever, visited by anglers. The sampled section starts at a small waterfall, which should prevent upstream migration under most conditions, and there is a weak but significant genetic differentiation between trout in the stream and the larger river below the waterfall (Taugbøl 2008).

The analyses described herein utilize empirical data described by Serbezov et al. (2010). Briefly, these were based on a total of 451 (195 males and 256 females) mature brown trout that were sampled (nondestructively) from Bellbekken during the 2002, 2003 and 2004 spawning seasons, and 1856 juvenile trout sampled the following years, aged 0+ (i.e., during their first year of life) to 2+ years and representing birth years or cohorts 2003 (834 individuals), 2004 (707) and 2005 (315). All fish were genotyped at 15 microsatellite loci. The genotypes were used to individually assign (with 90% confidence or higher) offspring individuals to potential parents, using the MasterBayes software (Hadfield et al. 2006). Altogether 935 of the 1856 sampled offspring were assigned a father and 723 were assigned a mother (Serbezov et al. 2010; table 3). Of the 451 potential parents, 78 males (of 195) and 116 females (of 256) were assigned one or more offspring to them, whereas 117 males and 140 females were not assigned with any of the sampled offspring. We combined parentage assignment and sib-ship reconstruction to improve our pedigree inferences as follows. The software COLONY v2.0 (Wang 2004; Jones and Wang 2010) was used to partition the offspring cohort into full and half-sib families and to infer their parental genotypes. For the 2002 breeding season, a few large offspring half-sib families had no matching parents among the sampled adults (Serbezov et al. 2010). These were assigned to generated parental genotypes and included in the calculations of the family-size variance as paternal half-sib families as they represent a large proportion of sampled offspring (Serbezov et al. 2010).

Bellbekken has poor growth conditions for brown trout and age determination based on scale reading is therefore uncertain. Genotype matching was used to quantify the error in age determination for adult fishes. Fish sometimes loose their Passive Integrated Transponder (PIT)-tags (Prentice & Flagg 1990), some of these individuals were tagged and genotyped again, and identical genotypes, combined with compatible length measurements, helped us confirm that an individual had been captured before. Specifically, we found that age was underestimated by 1 year in 35% and by 2 years in 18% of individuals that were 3 years or older. This uncertainty was incorporated when calculating generation times and modelling maturation probabilities, as described below. Ageing of juveniles was based on length and is thus not affected by errors in scale readings.

### Statistical analyses

**Figure 1:**
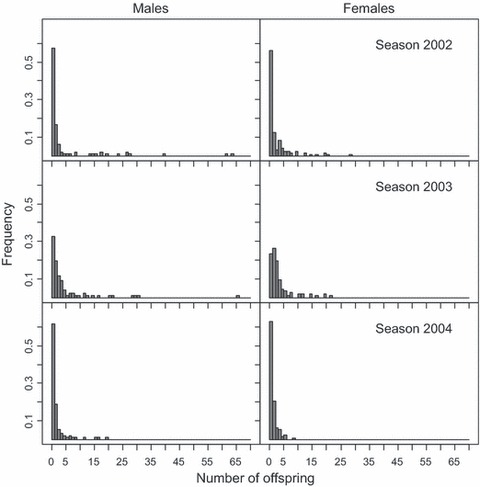
Observed distribution of reproductive success (number of progeny assigned) for male and female brown trout in Bellbekken during three consecutive spawning seasons.

We calculated the mean (*k*) and variances (*V*_k_) of offspring numbers per male and per female parent for each spawning season using data presented in Fig. 1. Because offspring were observed and counted when juvenile (age 0+ to 2+), and not at the same age as their parents, effects of mortality until reproductive age are not fully accounted for in these estimates of family size and their variances. To investigate the effect of family-specific mortality, we used the approach of Crow and Morton (1955) and adjusted *k* at different stages to their expected value of *k* = 2 (for a stable population) and recalculated *V*_k_. We calculated the mean (*k*) and variance (*V*_k_) of the number of progeny for each sex for 1-year-old offspring and for 3-year-old offspring separately. The age-specific ratios of variance to mean family size *R*_1_ (= *V*_k1_/*k*_1_) and *R*_3_ (= *V*_k3_/*k*_3_) values were then scaled to their expected values at *k* = 2 by assuming random survival from the last measured stage (Crow and Morton 1955; Waples 2002), and these are denoted as R*_1_ and R*_3_. This procedure yields an indication of how much family-size variance through a particular life stage has contributed to the reduction of the effective population size (using *N*_e_*/N* = *k*/(1 + R*_age_) (Waples 2002).

Calculation of *N*_e_ per generation requires an estimate of the variance in lifetime reproductive success (Hill 1972, 1979). As we only have estimates of reproductive success for single seasons, we used an individual-based simulation model for a population of 98 males and 108 females (the estimated average number of spawners in a spawning season for the 2002–2007 seasons, see below). We estimated maturation probability, *m*(*a*), at a given age, *a*, from the observed proportions, *o*(*a*), at that age (Barot et al. 2004):


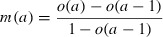
(1)

Uncertainties in observed age readings were included in the model by randomly sampling without replacement fish from the distribution of observed individuals and adding 1 or 2 years to the recorded age according to the observed error probabilities. This bootstrap technique was performed 1000 times. Because we found only marginal differences between estimates for males and females, we pooled data for both sexes (Fig. S1). The individual-based simulations were done by following individuals through their reproductive life, from 3 to 8 years of age, as all assigned parents were within these ages. Yearly reproductive success for mature fish was modelled by randomly sampling with replacement from the observed sex- and age-specific distributions of variation in reproductive success (Fig. 1). The number of offspring produced each year was then summed to give an individual estimate of lifetime reproductive success. Survival was modelled using the age-specific survival rate previously estimated based on capture–recapture estimates from our system (Carlson et al. 2008). This bootstrap technique was also performed 1000 times.

### Effective population sizes

We estimated effective population sizes per season (*N*_b_: effective number of breeders) and per generation (*N*_e_) from the seasons and lifetime distributions of family sizes, respectively. The effective numbers of breeders were calculated for each season and sex from the estimated mean number of offspring (*k*) and the corresponding variance (*V*_k_) (Table 1). For females, this was done using:


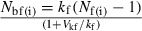
(2)

where *N*_fi_ is the estimated number of sexually mature females in a spawning season *i*, and *k*_f_ and *V*_kf_ are the mean and the variance of the number of progeny produced (Crow and Denniston 1988). The equation for male spawners is analogous. The number of sexually mature males and females in any given year was estimated using the results from the three-pass electrofishing results and the standard Zippin depletion method (Zippin 1958; Bohlin et al. 1989). To obtain confidence intervals for the estimated *N*_b(i)_’s, we resampled the observed number of spawners, with replacement, from their reproductive success. This bootstrap procedure was repeated 1000 times, and the 2.5% and 97% percentiles were taken as the lower and upper 95% confidence limits for *N*_b(i)_, respectively.

**Table 1:** Estimates of *N*_b_ for 2002–2004 in Bellbekken based on sex ratio and variance in the reproductive success (based on parentage assignment analysis). Shown are the estimated numbers of males and females, *N*_b_ is the number of breeders corrected for unequal sex ratio (after eqn (1)), *k*_m_, *V*_m_ and *k*_f_, *V*_f_ are the mean and the variance in reproductive success for males and females, respectively, and *N*_bm (i)_ and *N*_bf (i)_ are the respective effective numbers of males and females each season (after eqns 2 and 3)

Season	Males (*N*_m_)	Females (*N*_f_)	Males + females (*N*)	*N*_b_ (sex ratio)	*N*_b_ (sex ratio)/*N* (est)	*k*_m_	*V*_m_	*N*_bm (i)_	*k*_f_	*V*_f_	*N*_bf (i)_	*N*_bi_ (95% CI)	*N*_b_/*N*
2002	94	132	226	220	0.97	4.2	118.2	13.5	2.4	22.4	30.0	37.3 (26.2–49.3)	0.16
2003	85	103	188	186	0.99	4.8	94.0	19.6	2.9	18.0	40.7	52.9 (41.7–69.2)	0.28
2004	96	118	213	211	0.99	1.4	11.3	14.7	0.8	1.8	25.1	37.0 (28.2–45.6)	0.17
Average	91.7	117.7	209	205.7	0.98	3.5	74.5	15.9	2.0	14.1	31.9	39.8	0.20

An estimate of effective number of breeders in a year was obtained by combining female and male effective numbers for that year:


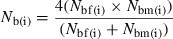
(3)

We also used the actual number of spawners (*N*_m_ and *N*_f_) in place of effective numbers in this eqn (3) to quantify the relative effects on *N*_e_ of unequal sex ratio alone.

The per-generation effective size, *N*_e_, was estimated from lifetime variances in family sizes obtained using the simulations described above and based on Hill’s (1972, 1979) approach, which is appropriate for species with overlapping generations. We use Hill (1979), eqn 10:


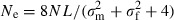
(4)

where *N* is the average estimated number of spawners (males plus females) in a season, *L* is the average generation length for males and females, and 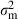 and 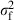 are the lifetime variances in reproductive successes for males and females, respectively. As presented by Hill (1972, 1979), calculations of these variances include separate variance and covariance terms for parent–offspring sex. As secondary sex characteristics are not developed until the fish approach maturity, offspring were not sexed in our study and we substituted 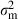 and 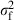 with *V*_km_ and *V*_kf_, respectively.

### Sensitivity analysis

To assess how the *N*_e_ estimates depend on the values of the model parameters, we re-analysed the model with varying parameter values. First, we ran the simulations using the age-maturation curve (Fig. S1) unadjusted for ageing errors. Then, we ran the simulations with a range of values for the male and female variance in reproductive success (0.5–10 times the observed value) and age-specific survival probabilities (from 40% less to twice the observed value).

### Cost of reproduction

We investigated the effect of previous reproductive effort on later reproductive success (i.e., cost of reproduction) from estimates of individual reproductive success over the three consecutive spawning seasons. The individual reproductive success in a given season may depend on sex, body length, age, identity of the spawner and year (because of e.g. environmental differences among years). Body length and age are highly correlated, and we used body length as covariate in the analysis because we have higher confidence in the precision of body length measurements than age (cf. ageing errors above). We use a generalized linear mixed effects model implemented in the R package MCMCglmm (Hadfield 2010) with the following structure to model effects on reproductive success:


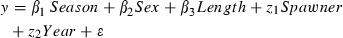
(5)

where *Season* (the number of the consecutive season an individual reproduces; from 1 to 3), *Sex* and fork *Length* are fixed effects, and *Spawner* identity and *Year* (2002, 2003, 2004) are random effects, and *ε* is random residual error. The response variable (*y*), specified in the model with Poisson distribution, is the estimated number of progeny produced per individual and year. Significant negative *β*_1_ values would be indicative of incurred costs of previous reproduction on current reproductive output.

## Results

Spawning brown trout found in Bellbekken were between 2 and 8 years of age. There was a tendency for females to mature slightly younger than males (mean age 5.8 vs 6.1 years; *t*_521_ = 3.9, *p* = 0.0006). The generation length of the two sexes, defined as their average ages at the time the offspring are born (the spring after a spawning event), was calculated from estimates obtained from the parentage data. The estimated generation time for males was 6.5 years (SD = 1.1 years) and for females 5.9 years (SD = 1.0), for an average of *L* = 6.2 years (SD = 1.1), in good agreements with estimates from other Scandinavian stream-resident brown trout populations (*L* = 6.2 – 6.6 years, Palm et al. 2003).

### Effective spawning population size

The estimated census number of breeders (*N*) in Bellbekken from 2002 to 2007 was relatively stable, ranging from 66 to 98 males and from 84 to 132 females. Based on these estimates, the effect on unequal sex ratios on *N*_b_ for 2002, 2003 and 2004 seasons was very small (eqn 3), and the *N*_b(sex ratio)_**/***N* ratios were negligibly different from unity in all three spawning seasons (Table 1).

In contrast, including the variances in family sizes (*V*_k_) from the parentage assignment analysis yielded (eqs 2 and 3) markedly reduced *N*_b_ estimates, with *N*_b_*/N* ratios in the range from 0.16 to 0.28 (Table 1, right). The impact of variance in reproductive success can be considered separately for each sex and spawning season by comparing effective numbers (*N*_bf_ and *N*_bm_) with census numbers (*N*_f_ and *N*_m_). From such comparisons, it is clear that deviations from binomial family-size distribution has a dramatic effect on reducing effective numbers for either sex (cf. Table 1), and the low *N*_b_/*N* ratios were not caused by a single sex only. This was observed in all three seasons and thus appears to be a general feature in this brown trout population.

Effects of among-family variation in survival on estimated lifetime means and variances in family sizes were assessed by the ratio of variance to mean progeny numbers (*R*_i_: Table 2), calculated separately for age 1 and 3 progeny. *R*_1_ values were always larger than 1, implying that family-size variances were larger than the binomial. This was more pronounced for males which, in addition to family-specific survival in the early juvenile stages, also reflect the reproductive skew in this population (see Serbezov et al. 2010). The adjusted R*_1_ values (indicating what the index of variability would be if survival was random from that point on to an adult population with *k* = 2) were in general smaller than the unadjusted *R*_1_ values, but still considerably larger than unity. The combined effects of all demographic processes, including among-family variation in juvenile survival, led to a further reduction (in most cases) of *N*_b_ relative to those presented in Table 1 and yielding final *N*_b_*/N* ratios between 0.14 and 0.41 (Table 2). The reduction in *N*_b_*/N* between the 1+ and 3+ stages was smaller relative to the reduction up to the 1+ stage, ranging from 32.41% further reduction (for females in 2002) to a 9.36% increase (females 2003). The mean family size at enumeration at the 3+ stage (between 0.4 and 1.4; Table 2) is less than what would be expected in a population of constant size, *k* = 2. The Crow and Morton (1955) approach for scaling *k* and *V*_k_, however, also applies if *k* < 2, the result being a scaled variance effective size that is equivalent to the inbreeding effective size that is calculated with the unscaled *k* and *V*_k_. All in all, the mortality between 1+ and 3+, although variable between sexes and seasons, does seem to be rather random among families, acting to reduce the negative effect of the strong skew in individual reproductive success on effective size and *N*_b_*/N* ratios.

**Table 2:** Demographic estimates used in analysing family-correlated survival. Shown are the estimated census spawner numbers (*N*), mean (*k*) and variance (*V*_*k*_) in family sizes for each sex and the corresponding age-specific index of variability (*R*_i_ = *V*_k/k_) and the scaled to their expected value at *k* = 2 age-specific index of variability (R^*^). These values are given for 2002 and 2003 and for the 1+ and 3+ age classes. The rightmost column shows the percentage change because of family-size variation that has occurred in the *N*_b_*/N* ratio between 1+ and 3+ stages

			1+ old fish					3+ old fish					
					
	Season	*N*	*k*_1_	*V*_1_	*R*_1_	R^*^_1_	*N*_b_/N = 2/1 + R^*^_1_	*k*_3_	*V*_3_	*R*_3_	R^*^_3_	*N*_b_/N = 2/1 + R^*^_3_	% Change between the two stages
Males	2002	94	3	57.7	19.4	13.4	0.14	1.4	12.3	9.1	13.0	0.14	−2.8
	2003	85	3.6	60.5	16.8	9.8	0.19	0.6	2.5	4	10.8	0.17	8.49
Females	2002	132	1.4	8.4	6.2	8.6	0.21	0.6	2.8	4.7	13.2	0.14	32.41
	2003	103	2.4	12.1	5	4.4	0.37	0.4	0.6	1.6	3.9	0.41	−9.36

### Repeat spawning

**Figure 2:**
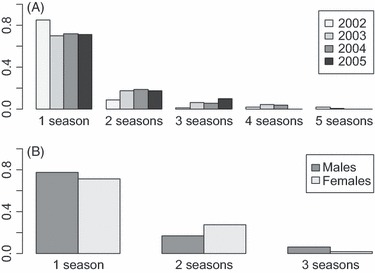
(A) Proportions of brown trout individuals that were observed to have been sexually mature during different consecutive breeding seasons (1–5 seasons). The four categories (2002–2005) denote the first year a spawner was observed to be sexually mature. (B) Proportions of assigned spawners of both sexes in 1–3 consecutive seasons.

A third of the spawners in Bellbekken were observed to be sexually mature during more than one season; a few individuals of each sex were even observed to be mature during five consecutive seasons (Fig. 2A). There was no difference between the sexes in the proportion of iteroparous (repeat spawning) individuals (χ^2^ = 0.68, df = 1, *p* = 0.41) or in the levels of realized iteroparity (being assigned offspring in more than one season) (χ^2^ = 0.94, df = 1, *p* = 0.33) (Fig. 2B). On average, iteroparous males increased their reproductive success by 52% and females by 38% relative to semelparous fish. The only parameter from the mixed linear effect model (eqn 5) that had significant effect on reproductive success was body length (mean ± SD: 0.09 ± 0.02). There seem to be quite large among-year variation in the number of offspring (mean ± SD: 21.20 ± 58.49) that masks any possible effects of previous reproduction on the current offspring production, and no cost of reproduction was detected.

### Per-generation effective size

Using the lifetime variances in estimated family sizes (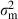 = 82.6 and 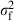 = 22.7), *L* = 6.2 (the average generation length) and *N* = 196 (average estimated number of spawning individuals for the 2002–2007 period) in Hill’s eqn (4), *N*_eV_ was estimated to be 104.3 ± 44.9 (SD), and a *N*_eV_*/N* ratio of 0.53 ± 0.24 (SD).

**Figure 3:**
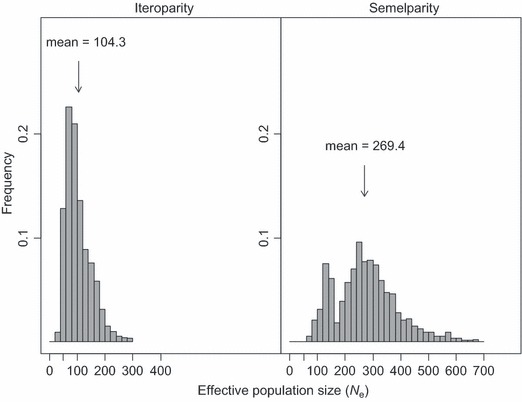
Distribution of simulation-based estimates of *N*_eV_ for the iteroparous model (A) and the semelparous model (B). The lifetime variance in reproductive success is simulated based on sex- and age-specific fertilities from brown trout in Bellbekken.

To evaluate the consequences of repeat spawning on effective size in brown trout, we introduced obligate mortality (semelparity) after a maturation event in the model. This had the effect of reducing the number of offspring per breeder substantially for both sexes (3.8 ± 8.8 vs 1.8 ± 5.2 for males, *t*-test: *p* < 0.0001, 2.7 ± 4.7 vs 1.8 ± 3.7 for females, *t*-test: *p* < 0.0001), but also reduced its variance (*F*-test to compare two mean-standardized variances: *F*_1,13460_ = 2.8, *p* < 0.0001 for males; *F*_1,13480_ = 1.6, *p* < 0.0001 for females). This reduction in variance in family size resulted in a *N*_eV_ estimate for the hypothetical semelparous situation of 272.0 (SE = 112.7), more than twice the estimate with repeat spawning (Fig. 3). The increased family variance however led to a increased variance (uncertainty) in the *N*_eV_ estimate (*F*-test to compare two mean-standardized variances: *F*_1,999_ = 5.7, *p* < 0.0001).

### Sensitivity analysis

**Figure 4:**
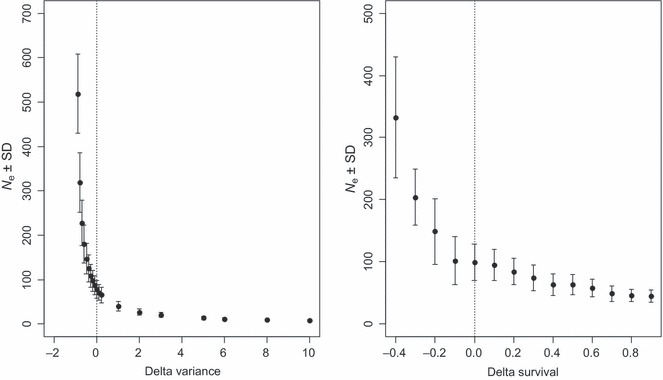
Sensitivity of the *N*_e_ estimates to changes (% change from the observed values, vertical dashed line) in reproductive success variance (A) and age-specific survival estimates (B).

The *N*_eV_ estimates were relatively sensitive to changes in the variance in reproductive success, especially at lower values compared to the obtained values (Fig. 4A), but very little with respect to the value of the age-specific survival estimates (Fig. 4B). The *N*_eV_ estimates were also rather insensitive to the shape of the age-specific probability of maturation curve (Fig. S1), giving slightly higher estimate when using the age-determination-error unadjusted maturation probabilities (128.1 ± 51.2).

## Discussion

In this study of an exhaustively sampled stream-resident brown trout population, we were able to estimate effective number of spawners per season and generation and to quantify several factors that impacted the *N*_b_*/N* and *N*_e_*/N* ratios. This is the first study to our knowledge that uses parentage genetic assignments data to estimate effective population sizes in a natural salmonid population, yielding separate estimates for per season and per generation. Earlier attempts on this species have relied on temporal change in allele frequencies, yielding only per-generation estimates (Jorde and Ryman 1996; Laikre et al. 1998, 2002; Hansen et al. 2002; Palm et al. 2003; Heggenes et al. 2009). While these approaches to estimate per-generation *N*_e_ yielded congruent results, our approach has the great advantage of also providing estimates of per season *N*_b_ and of the factors determining effective sizes. As expected, the effect of the variance in reproductive success led to the largest reduction in *N*_e._ relative to the census size (*N*), whereas unequal sex ratios had only a marginal effect. The impact of family-size variation might be buffered with age as juvenile survival seems to be rather random at older juvenile stages with respect to families. Simulation results indicate that iteroparity, on the other hand, increases the variance in reproductive success, leading to a decreased effective population size. Iteroparity however also decreased the variance of the effective population size, which might lead to a decreased risk of demise because of stochastic population events.

The genetic parentage assignments and the exhaustive sampling should allow for robust estimates of *N*_b_. Nevertheless, nearly half of the offspring were not assigned a parent, and there was some uncertainty in the ageing of mature fish, which might bias the generational *N*_e_ estimate. We do not expect that the high proportion of unsampled parents would have significant impact on the 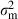 and 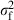, as there is no reason to suspect that the unsampled spawners produce a different family-size distribution. Also, the *N*_e_ estimate seemed to be insensitive to ageing errors as analyses done without making age adjustments (see Materials and methods section) yielded estimates that were only slightly higher. The *N*_e_ estimate also seems to be quite insensitive to higher family-size variance and survival values than the observed ones, but increase significantly at lower variance values (Fig. 4). This result is expected for the family-size variance, whereas decrease in survival seems to increase family-size variance and thus to lower generational *N*_e_ value.

We arrive at *N*_b_*/N*_i_ estimates of 0.16–0.28, whereas the *N*_e_*/N* value was around 0.5 from our simulation model. Because census size is often the only available demographic parameter from wild populations, obtaining taxon-specific *N*_b_*/N*_i_ and *N*_e_*/N* would be useful for monitoring changes in genetic diversity. The *N*_b_*/N*_i_ values are within the range of those reported for other salmonids, both semelparous (0.1–0.4, (Hendry and Stearns 2004)) and iteroparous (0.11–0.31, (McLean et al. 2007)). Generational *N*_e_*/N* is theoretically expected to be around 0.5 for iteroparous species with overlapping generations (Nunney 1993) and range between 0.25 and 0.75 (Nunney and Elam 1994; Waite and Parker 1996). In a recent review, Palstra and Ruzzante (2008) reported a median *N*_e_*/N* value of 0.14 for a range of organisms, but that this ratio is actually higher for small populations. The formula for effective size of the population assumes no environmental stochasticity, stable age structure and constant population size. However, in organisms with overlapping generations, *N*_e_*/N* ratios below 0.1 apparently require extreme conditions, one of which might be selection (Nunney 1993, 1996; Nunney and Elam 1994). Comparisons of estimated *N*_b_ (39.8, on average) and *N*_e_ (104) verifies that for iteroparous species *N*_e_ per generation is not simply a product of per season *N*_b_ and generation length (approximately 6 years), as is the case for semelparous species (Waples 1990).

### Family-specific survival

Survival at later juvenile stages (older than 1+) was rather random with respect to families and occasionally led to increases in *N*_b_*/N*_i_ (Table 2). Similar pattern has also been found in other salmonid studies (Hedrick et al. 2000). More pronounced family-correlated survival might be expected for younger juvenile stages than what we can make inference for, when the juveniles belonging to a sib-ship group are especially vulnerable and tend to be spatially clumped (Serbezov et al. 2010). We observed, however, marked year-to-year variation in the amount by which *N*_b_*/N* is reduced between 1+ and 3+ life stages. Stochastic environmental or demographic events would explain such year-to-year variation. Year-to-year variability in juvenile survival has been shown to dominate over size-dependent survival in this system (Carlson et al. 2008).

Our simulation results show that the iteroparous system has higher variance in reproductive success that consequently lowered the *N*_e_ value. This was despite the fact that iteroparous individuals are more productive because of higher cumulative fecundity and lifetime fitness (see also Fleming and Reynolds 2004). In polygamous systems, iteroparity can be thought to lead to subordinate individuals having more opportunities to be successful, reducing the between-individual variance in reproductive success and thus increasing *N*_e_ (Nunney 1993). We could not find any significant cost of reproduction, but our results revealed that the effect of body size on siring offspring is significant (see also Serbezov et al. 2010). Larger individuals leave more offspring compared to smaller individuals, and this difference widens as individuals reproduce repeatedly. Such a scenario conforms with the fact that family-size variance increases with decrease in survival in our sensitivity analysis. Other studies have also conjectured that iteroparity and multiple paternity can increase the variance in reproductive success and therefore reduce *N*_e_ (Karl 2008). In the closely related Atlantic salmon (*Salmo salar*), repeat spawners can also produce disproportionate share of recruitment (Mills 1989).

Small populations may fluctuate in numbers for a variety of reasons, including demographic stochasticity, in addition to natural or human-driven environmental perturbations. If population fluctuations are sufficiently large and not rapidly attenuated, they could lead to loss of genetic diversity and possibly population extinction (McCauley 1991). Complexity, roughly defined as the degree of iteroparity, is positively correlated with stability in terms of ability to recover from perturbations (Demetrius et al. 2004). Here, the *N*_e_ estimates obtained by assuming strict semelparity led to increased variance in the *N*_e_ estimates, implying that such a population might be more exposed to stochastic demographic processes. There were signs of demographic/genetic stochasticity/perturbations in our system. The 2005 cohort appears to be relatively much reduced in samplings from fall 2006, despite similar sampling effort and catchability (data not shown). The estimated total number of individuals in the stream varied up to 45% between the seasons investigated. This considered, and in the light of the size of the population, demographic stochasticity might not be uncommon in this system. Nevertheless, the system seems to be resilient to perturbations as it shows relatively high levels of genetic variation (data not shown). Iteroparity, which can be viewed as a ‘bet-hedging’ strategy to deal with environmental uncertainty, thus might be an effective strategy in such a system which might regularly experience years with poor recruitment and low number of breeders (Gaggiotti and Vetter 1999). Some support for this comes from empirical studies on long-lived fish species (e.g., Diaz et al. 2000; Lippe et al. 2006), which reported the maintenance of high genetic diversity despite relatively small population sizes. Patterns of iteroparity between steelhead trout (*Oncorhynchus mykiss*) populations reveal that the levels of iteroparity are highest at latitudinal extremes and suggest that it might be a more beneficial strategy at harsher/less predictable conditions (Stearns 1976; Roff 2002; Einum and Fleming 2007).

From a conservation or management standpoints, it is important to understand how populations might evolve in response to environmental changes and how these responses might then feed back on population persistence and productivity. Among other things, this requires an understanding of the factors affecting effective population size. Here, our results emphasize the importance of obtaining knowledge of important life history traits and delineating their impact on evolutionary dynamic processes such as those reflected by *N*_e_. The life history and demographic parameters that are most essential to a population’s conservation can thus be better identified, especially in species with complex life histories, allowing researchers to design improved management and conservation programmes. Namely, we documented large effect of the variance in reproductive success on reducing the *N*_b_*/N*_i_ ratio in a wild salmonid population. However, this reduction may be buffered with age of the juveniles as family-specific survival seems to be rather random at older juvenile stages. Simulation results also indicated that iteroparity increases the variance in reproductive success, leading to a decreased effective population size. At the same time, iteroparity decreases the variance of the effective population size, which might lead to a decreased risk of demise because of stochastic population events. The evolutionary implications of effective population size therefore depend on detailed knowledge of its interactions with life history and population dynamics. These insights may be attained only through carefully considering age structure in empirical investigations of *N*_e_ (Waples 2010). Finally, environmental change might produce conflicting selection pressures in different life stages in organism with complex life histories, which will interact with plastic (i.e., nongenetic) changes in various ways (Crozier et al. 2008). In the case of the resident brown trout population studied here, we infer high levels of resilience, which results from a combination of plastic life history traits such as promiscuous mating system and iteroparity. Specifically, the *N*_e_ seems to be pretty resilient with respect to age-specific maturation probabilities and survival rates. Nevertheless, the factors identified in this paper to reduce the *N*_e_ should be carefully considered in conservation and management applications.

## Data archiving statement

The raw data used for the parentage assignment analyses were archived for Serbezov et al., Molecular Ecology 19:3193–3205 and can be found at Dryad: doi:10.5061/dryad.1597.

## Supporting Information

**Figure S1.** Logistic regression relating brown trout age and the probability of being mature at that age in Bellbekken.
