# Erratum

**DOI:** 10.1111/eva.12035

**Published:** 2012-12-24

Paper to correct:

Serbezov, D. et al. 2012. Life history and demographic determinants of effective/census size ratios as exemplified by brown trout (Salmotrutta). Evolutionary Applications. doi: 10.1111/j.1752/4571.2012.00239.x

Erratum:

Palstra et al. 2009 was unfortunately omitted from the following sentences in the Discussion. The corrected sentences are:

– ‘Iteroparity, which can be viewed as a ‘bet-hedging’ strategy to deal with environmental uncertainty, thus might be an effective strategy in such a system which might regularly experience years with poor recruitment and low number of breeders (Gaggiotti and Vetter 1999, Palstra et al. 2009)’.

– ‘The evolutionary implications of effective population size therefore depend on detailed knowledge of its interactions with life history and population dynamics. These insights may be attained only through carefully considering age structure in empirical investigations of Ne (Palstra et al. 2009, Waples 2010)’

Omitted Reference: Palstra, F. P., M. F. O'Connell, and D. E. Ruzzante. 2009. Age structure, changing demography and effective population size in Atlantic salmon (Salmosalar). Genetics 182:1233–1249 doi: 10.1534/genetics.109.101972.x

