# Supplementary figures and images for: Life history and demographic determinants of effective/census size ratios as exemplified by brown trout (Salmo trutta)
Source: Evol Appl. 2012 Jan 23;5(6):607–18. doi: 10.1111/j.1752-4571.2012.00239.x (PMC3461143; doi:10.1111/j.1752-4571.2012.00239.x)

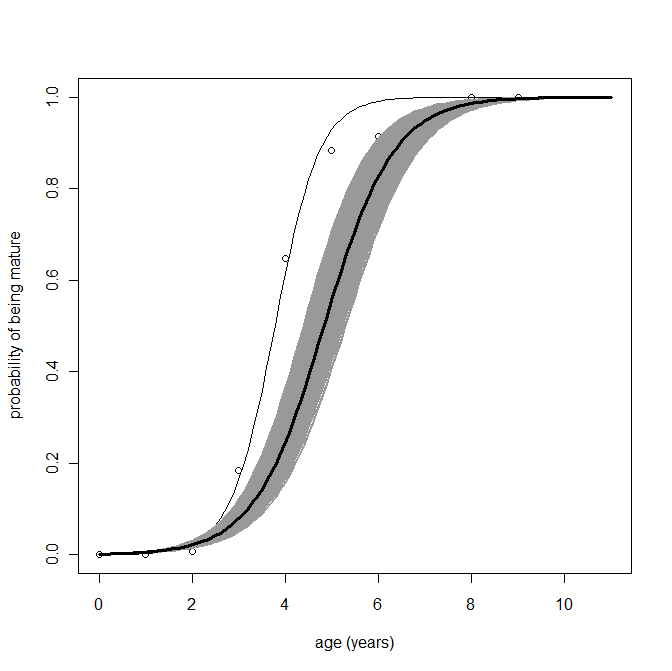


Figure S1.

Supplement: Supplementary file 1 [file eva0005-0607-SD1.doc]
