# Supplementary material for: Life history and demographic determinants of effective/census size ratios as exemplified by brown trout (Salmo trutta)
Source: Evol Appl. 2012 Jan 23;5(6):607–18. doi: 10.1111/j.1752-4571.2012.00239.x (PMC3461143; doi:10.1111/j.1752-4571.2012.00239.x)
Supplement: Supplementary file 2 [file eva0005-0607-SD2.pdf]

Appendix A: Figure showing the predicted probabilities of being mature at a given age

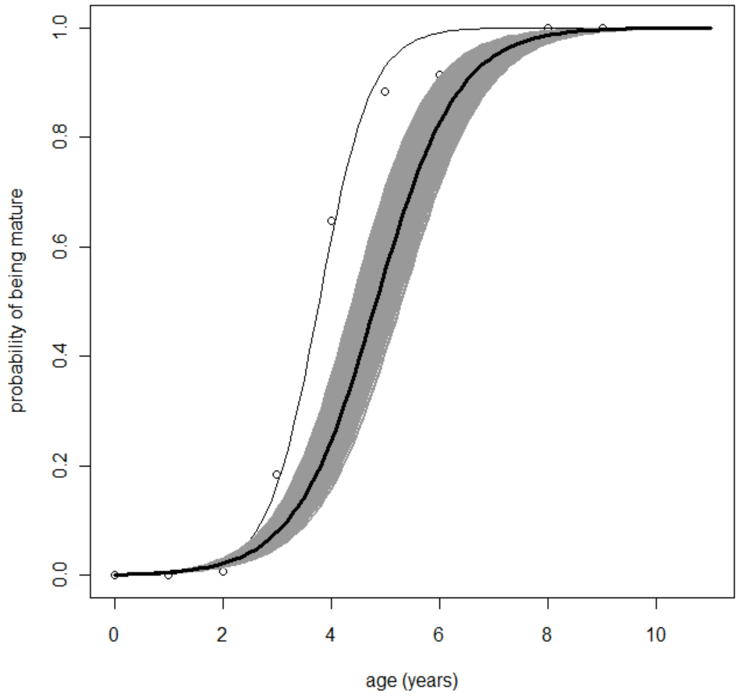

Figure A1. Logistic regression relating brown trout age and the probability of being mature at that age in Bellbekken. The thin black line is the model calculated based on the observed data, and the thick line is based on age-determination-error adjusted age. Data are pooled for all years and both sexes and the grey envelope marks the distribution based on 100 simulations.
